# Reference Range of Quantitative MRI Metrics Corrected T1 and Liver Fat Content in Children and Young Adults: Pooled Participant Analysis

**DOI:** 10.3390/children11101230

**Published:** 2024-10-12

**Authors:** Elizabeth Shumbayawonda, Cayden Beyer, Benito de Celis Alonso, Silvia Hidalgo-Tobon, Briceida López-Martínez, Miguel Klunder-Klunder, América Liliana Miranda-Lora, E. Louise Thomas, Jimmy D. Bell, David J. Breen, Kamil Janowski, Maciej Pronicki, Wieslawa Grajkowska, Malgorzata Wozniak, Elzbieta Jurkiewicz, Rajarshi Banerjee, Piotr Socha, Po-Wah So

**Affiliations:** 1 Perspectum Ltd., Oxford OX4 2LL, UK; 2 Faculty of Physical and Mathematical Sciences, Benemérita Universidad Autónoma de Puebla, Puebla 72000, Mexico; 3 Imaging Department, Children’s Hospital of Mexico Federico Gómez, Mexico City 06720, Mexico; 4 Physics Department, Universidad Autónoma Metropolitana, Campus Iztapalapa, Mexico City 09340, Mexico; 5 Sub Direction of Research, Children’s Hospital of Mexico Federico Gómez, Mexico City 06720, Mexico; 6 Research Committee, Latin American Society for Pediatric Gastroenterology, Hepatology and Nutrition (SLAGHNP/LASPGHAN), Mexico City 06720, Mexico; 7 Epidemiological Research Unit in Endocrinology and Nutrition, Children’s Hospital of Mexico Federico Gómez, Mexico City 06720, Mexico; 8 Research Centre for Optimal Health, University of Westminster, London W1B 2HW, UK; 9 Department of Radiology, University Hospital Southampton NHS Foundation Trust, Tremona Road, Southampton SO16 6YD, UK; 10 Department of Gastroenterology, Hepatology, Nutritional Disorders and Pediatrics, The Children’s Memorial Health Institute, 20 04-736 Warsaw, Poland; 11 Department of Pathology, The Children’s Memorial Health Institute, 20 04-736 Warsaw, Poland; 12 Department of Diagnostic Imaging, The Children’s Memorial Health Institute, 20 04-736 Warsaw, Poland; 13 Department of Neuroimaging, Institute of Psychiatry, Psychology and Neuroscience, King’s College London, London SE5 8AF, UK

**Keywords:** fibro-inflammation, multiparametric MRI, noninvasive, paediatric, reference ranges

## Abstract

Background: Multiparametric MRI markers of liver health corrected T1 (cT1) and proton density fat fraction (PDFF) have shown utility in the management of various chronic liver diseases. We assessed the normal population reference range of both cT1 and PDFF in healthy child and adult volunteers without any known liver disease. Methods: A retrospective multi-centre pooled analysis of 102 child and young adult (9.1 years (6–18)) volunteers from three centres: Children’s Memorial Health Institute (N = 21), University Hospital Southampton (N = 28) and Hospital Infantil de Mexico (N = 53). Sex and ethnic differences were investigated for both cT1 and PDFF. Age effects were investigated with comparison to a pooled adult cohort from the UK Biobank (N = 500) and CoverScan (N = 71), covering an age range of 21 to 81 years. Results: cT1 values were normally distributed with a median of 748 ms (IQR: 725–768 ms; 2.5–97.5 percentiles: 683–820 ms). PDFF values followed a normal distribution with a median of 1.7% (IQR: 1.3–1.9%; 2.5–97.5 percentiles: 1–4.4%). There were no significant age and sex differences in cT1 and PDFF between children and young adults. No differences in cT1 and PDFF were found between ethnicities. Age comparisons showed statistically significant, but clinically negligible, cT1 (748 ms vs. 732 ms) and PDFF (2.4% vs. 1.9%) differences between paediatric and adult groups, respectively. Conclusions: Median healthy cT1 and PDFF reference ranges in children and young adults fall within the reported limits for normal of 800 ms and 5%, respectively.

## 1. Introduction

Paediatric liver disease is rapidly becoming one of the major causes of premature mortality in children and young adults [1,2]. Although there is increasing awareness linking excessive liver fat to other potentially chronic comorbidities such as type 2 diabetes mellitus [3], dyslipidaemia, hypertension, cardiovascular disease and renal disease [4], the prevalence of obesity has increased the most rapidly over the past decade [5,6]. In the United States, studies have shown prevalence rates of 46% for metabolic dysfunction-associated liver disease (MASLD) and 12% for metabolic dysfunction-associated steatohepatitis (MASH) in this young population [7]. Unfortunately, due to its asymptomatic nature or sometimes nonspecific presentation, liver disease frequently goes undetected, resulting in increased hazard ratios for the development of primary liver cancers (including hepatocellular carcinoma [HCC] and cholangiocarcinoma [CCA]) [8] and overall cardiometabolic-specific mortality compared to matched general population controls [2].

Although the use of noninvasive technologies (NITs) to support clinical management is not a new concept, the validation and adoption of these NITs in paediatric management to provide equivalent support in this population as seen in adult care is an area of unmet need [9]. For instance, the use of ultrasound is typically not recommended in children and young adults for the determination or quantification of steatosis due to poor sensitivity and specificity [9,10]. In addition, the use of computed tomography (CT) is not recommended in this population due to radiation risk [9].

Multiparametric magnetic resonance imaging (mpMRI) of the liver has been shown to have prognostic capabilities in the paediatric chronic liver disease setting and the detection of treatment response [11]. Corrected T1 (cT1), a mpMRI marker of fibro-inflammatory disease activity, is sensitive to subtle changes in liver tissue composition, and increases with the histological stage of fibrosis and inflammation in both adults and paediatrics [12]. Alongside being an independent predictor of inflammation in children and young adults with autoimmune liver diseases [13], cT1 has shown good clinical utility for the identification of ongoing active disease despite biochemical remission [14]. In Fontan-associated liver disease, cT1 was significantly associated with reduced exercise capacity and increased levels of liver fibrosis/congestion [15]. cT1 has also shown utility in identifying the presence of radiologically detected portal hypertension in chronic progressive paediatric autoimmune liver diseases with high sensitivity and specificity [16,17] and radiologically detected and clinical biomarkers of liver fibrosis [18]. In addition to being validated against biopsy in both adult and paediatric populations, cT1 is standardised across multiple scanners and field strengths. Therefore, cT1 is advantageous over conventional T1 or ultrasound as the metrics can be compared objectively across populations and longitudinally within individuals.

As cT1 [19] and PDFF [4] values are already validated in healthy adult populations and reference ranges exist, the aim of this study was to provide a set of reference values for corrected T1 within a population of nominally healthy participants of varying ages who are at nominally low risk of chronic liver disease. To this end, we conducted a multi-centre pooled individual data analysis to describe the range of cT1 and liver fat (proton density fat fraction; PDFF) values in a population of children and young adults. We also used data from two studies in adults to investigate if there are any age effects on cT1 and PDFF values.

## 2. Methods

### 2.1. Study Population

This was a retrospective study of MR data acquired as part of five studies (three in children and young adults and two in adults). Individual participant data for healthy child and young adult (paediatrics) volunteers scanned as part of the Kids4LIFe study (NCT03198104), which received ethical approval (11/KBE/2016) in Poland and the United Kingdom (16/SC/0621), were included in the analyses. Additionally, healthy asymptomatic volunteers from the METCOG study, which received ethical approval from the King’s College London Psychiatry, Nursing and Midwifery Research Ethics Subcommittee (RESCM-18/19-4156) and the Hospital Infantil de Mexico, Federico Gómez (HIM/2016/105 and SSA-1369), were also included in the analysis as part of the paediatric group. In addition to the respective inclusion and exclusion criteria from each of the studies, for the paediatric group, ‘healthy’ was defined as having a BMI within the 5th to 85th percentiles using the Centre for Disease Control and Prevention (CDC) guidelines for children and teenagers, normal liver function test results (ALT and AST < 40 IU/L), and no known or previously diagnosed chronic liver disease.

Adult data were acquired from the UK Biobank (UKBB) imaging enhancement study between 4 January 2016 and 2 February 2020. For adults, ‘healthy’ was defined as having PDFF ≤ 5%, BMI ≤ 25 kg/m^2^, weekly alcohol consumption < 14 units, normal liver function tests (ALT and AST < 40 IU/L), no diabetes and no hypertension. However, as the UKBB generally includes much older participants, to ensure that a representative age range was covered, healthy controls from the CoverScan study (NCT04369807, ethics reference: 20/SC/0185) were also included as part of the adult sub-group. 

### 2.2. Imaging Protocol and Post-Processing

Imaging acquisition for the Kids4LIFe study took place at the Children’s Memorial Health Institute (IPCZD) using a 1.5T Siemens Avanto systems scanner (Siemens Healthineers, Erlangen, Germany) and a 3T Siemen’s Skyra scanner (Siemens Healthineers, Erlangen, Germany) at University Hospital Southampton. For the METCOG study, MR images were acquired on a 3T Siemen’s Skyra scanner (Siemens Healthineers, Erlangen, Germany) at Hospital Infantil de México Federico Gómez. UKBB images were acquired from participants who were scanned at one of four UK Biobank imaging centres (in Newcastle upon Tyne, Stockport, Reading and Bristol) on Siemens Aera 1.5 T scanners (Siemens Healthineers, Erlangen, Germany). MRI images from healthy volunteers who were part of the CoverScan study (serving as the control group) were obtained on either an Aera 1.5T or Vida 3T Siemens MAGNETOM scanner (Siemens Healthineers, Erlangen, Germany).

All mpMR images were obtained using the LiverMultiScan (Perspectum Ltd., Oxford, UK) image acquisition protocol with MRI scanning sequences reported previously [20]. For the Kids4Life, METCOG and CoverScan studies, four transverse slices obtained at the porta hepatis location in the liver were acquired for each participant using a shortened modified look-locker inversion (shMOLLI) and a multi-echo spoiled gradient-echo sequence to quantify T1 [21]. For the UKBB study, to meet the high throughput demands of the study (resulting in short acquisition times of ≤3 min), a single transverse slice, located at the porta hepatis, was used to quantify liver metrics. Both approaches have been shown to correlate well with histology and predict both liver and cardiac-related outcomes [14,22,23].

**Figure 1:**
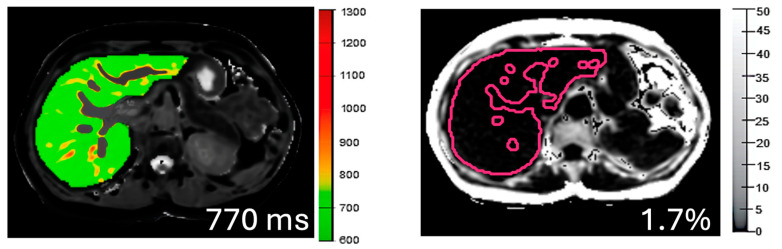
Whole liver segmentation cT1 and PDFF maps of the liver. In the cT1 maps, lower values (cooler colours in maps and colour bar) represent areas with lower cT1 values and therefore lower disease activity, while higher cT1 values (warmer colours) would represent areas of the liver with active disease. In the PDFF maps, lower values (darker) represent lower liver fat values, whilst lighter shades would represent higher liver fat.

During image analysis, iron-corrected T1 (cT1) and PDFF maps of the liver were delineated into whole liver segmentation maps using a semi-automatic method. Three 15 mm diameter circular regions of interest were placed on the transverse T2* maps for each slice, covering a representative sample of the liver, to calculate average T2* values for T1 correction. All images were analysed by trained analysts (technologists) blinded to the clinical data. During analysis, non-parenchyma structures such as bile ducts and large blood vessels as well as image artifacts are automatically excluded (Figure 1).

### 2.3. Statistical Analysis

Descriptive statistics were used to summarise cohort characteristics. Summary data are presented as either medians with interquartile ranges (IQRs) or means with standard deviation (SD). Kruskal–Wallis tests were used to test for statistically significant differences between sexes (males and females) and ethnicities. Participants in the paediatric group were further classified as either a child (aged 6–12 years) or young adult (aged 13–18 years). To further evaluate the differences between the paediatric and adult populations, the adult group was classified as adult (aged 19–60 years) or older adult (aged > 60 years). A two-sample *t*-test was used to compare between age sub-groups.

Lower and upper thresholds of the reference ranges were obtained from the data based on the mean ± 1.96 × SD for data that were normally distributed. The 2.5th and 97.5th percentiles were also provided to illustrate the distribution of the data.

Inter- and intra-reader variability in cT1 and PDFF analysis was determined in the sub-set of paediatric data collected from the Kids4LIFe study. Two technologists analysed the anonymised datasets on day one and then again on day 30. Intra- and inter-reader agreement was calculated using intra-class correlation (two-way mixed model with fixed effects) and Bland–Altman analyses. Two sets of Bland–Altman analyses were performed. The first set compared values acquired on day one and day 30 for each of the analysts. To determine the intra-rater reliability, the mean and range of the limits of agreement from the Bland–Altman analyses were calculated. The second set of Bland–Altman analyses was an all-against-all analysis of data from day one between both analysts. To demonstrate the inter-rater reliability, the mean and range of the limits of agreement from these Bland–Altman analyses were calculated. All statistical analysis was performed using R version 4.2.2 (R Core Team, Vienna, Austria), with values of *p* < 0.05 considered statistically significant.

## 3. Results

### 3.1. Demographics

N = 101 healthy child and young adult (paediatric) volunteers were included in the analysis (median age 9.1 years (aged 6.0–18.3), with 72% male) (Table 1). As a comparator, an adult group of N = 571 was included with a median age of 63 years (aged 21.0–81.0, 43% male).

### 3.2. cT1 Distribution

**Figure 2:**
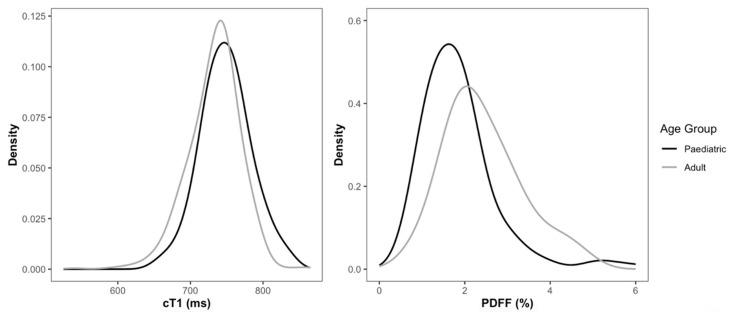
Distribution of cT1 and PDFF values for healthy adult and paediatric subgroups. Paediatric participants were aged 6–18 years, whilst adult participants were aged 21–81 years.

In the paediatric subgroup, cT1 values were normally distributed, with a median value of 748 ms (IQR: 725–768 ms; 2.5th–97.5th percentiles of 683–820 ms) (Table 2). Similarly, in the adult subgroup, cT1 values were normally distributed, with a median value of 738 ms (IQR: 714–754 ms; 2.5th–97.5th percentiles of 654–791 ms) (Table 2). Although numerically similar, with a median difference of <20 ms, cT1 groupwise comparisons between adult and paediatric subgroups showed significant differences between the two groups (*p* < 0.001) (Figure 2 and Table 3).

**Figure 3:**
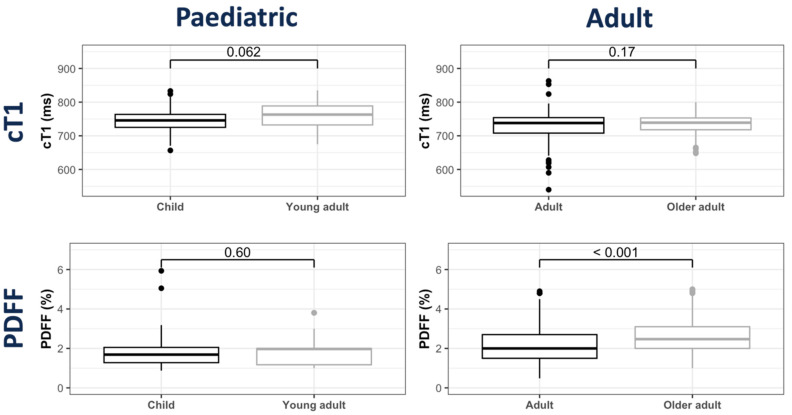
Box plots illustrating groupwise distribution of cT1 and PDFF between age sub-groups. Age groups for healthy participants were defined as follows: Child aged 6–12 years, Young Adult aged 13–18 years, Adult aged 21–60 years and Older Adult aged 61–81 years.

As the paediatric subgroup covered a wide range of ages, including both pre- and post-puberty, further investigations were performed after the classification of participants into two sub-groups (child and young adult). Groupwise comparisons showed no significant differences in cT1 (*p* = 0.062) between children and young adults (Supplementary Materials Table S1). Further explorations into the differences in cT1 due to age showed no significant differences between children and young adults or adults and older adults (Figure 3 and Supplementary Materials Table S1). Comparisons across all age sub-groups showed significant differences between the child, adult and older adult sub-groups, as well as between the young adult, adult and older adult sub-groups (Supplementary Materials Table S1 and Figure S2). However, similar to the previous comparisons, the age sub-group comparisons were statistically significant, but numerically similar, with a median difference of <20 ms.

### 3.3. PDFF Distribution

In the paediatric subgroup, PDFF values followed a normal distribution and had a median value of 1.7% (IQR: 1.3–2.1%; 2.5th–97.5th percentiles of 1.0–4.4%) (Figure 2 and Table 2). Similarly, in the adult subgroup, PDFF values were also normally distributed, with a median value of 2.3% (IQR: 1.8–3.0%; 2.5th–97.5th percentiles of 0.9–4.6%). Although both values were within the normal ranges reported for healthy individuals, groupwise comparisons between adult and paediatric subgroups showed statistically significant, but clinically negligible, differences between the two groups (*p* < 0.001), with adults having higher PDFF (2.4% ± 0.9) compared to paediatrics (1.9% ± 0.9) (Table 3).

Groupwise comparisons between the child and young adult sub-groups showed no significant PDFF differences (Figure 3 and Supplementary Materials Table S1). Further comparisons across all age groups highlighted a statistically significant, but clinically negligible, increasing trend in PDFF with age (Supplementary Materials Table S1 and Figure S2).

### 3.4. Sex and Ethnicity Characteristics

Table 3 shows the distribution of cT1 and PDFF in the paediatric group between males and females. Overall, the effects of sex were minimal and statistically insignificant for both cT1 (*p* = 0.268) and PDFF (*p* = 0.452). An investigation of sex differences in the children and young adult subgroups also showed no significant groupwise differences (Table 3 and Supplementary Materials Table S1). Further sex groupwise comparisons showed significant differences (*p* = 0.019) in cT1 in only the adult subgroup (Supplementary Materials Table S1). Although statistically significant sex differences between paediatric and adult groups were observed for PDFF (*p* < 0.001), the numerical difference (0.3 percentage points) was negligible (Table 3). Further PDFF groupwise comparisons showed statistically significant, but clinically negligible, differences between males and females in the adult and older adult subgroups. 

The paediatric group was made up of Hispanic and Caucasian participants; investigations were performed to evaluate any ethnic differences between the groups. Included in this investigation was the adult group, which was comprised of mainly the Caucasian (95%) ethnicity, among others (Chinese, Indian, Caribbean, Pakistani, Bangladeshi and other mixed ethnic groups). Findings showed that there were no significant cT1 differences between ethnic subgroups (Supplementary Materials Table S1). PDFF comparisons showed numerical statistically significant differences between ethnicity subgroups, with the Caucasian subgroup having the highest PDFF values; however, these were clinically negligible (Supplementary Materials Table S1).

### 3.5. Technical Performance: Inter- and Intra-Reader Variation in cT1 and PDFF Assessment

Inter and intra-rater reproducibility for cT1 and PDFF readings is summarised in Table 4. For both cT1 and PDFF, the inter-class correlation indicated excellent agreement between raters (technologists) and there was minimal bias and narrow 95% limits of agreement (cT1: mean difference 30 ms [95% CI ± 18 ms]; PDFF: mean difference 1.9% [95% LoA: ±1.0%]). The magnitude of the largest bias between raters (technologists) was 7 ms for cT1 and 0.3% for PDFF (Table 4 and Supplementary Materials Figure S1).

The intra-class correlation for individual technologists analysing cT1 data on different days was 0.99 (95% CI 0.99–1.00), with a mean difference between the upper and lower limits of agreement of 1 ms (with limits of agreement of −2 ms to 0 ms based on the Bland–Altman plot) (Supplementary Materials Figure S1). The mean upper and lower limits of agreement for cT1 were 8 ms (range: 4 to 11 ms) and −18 ms (range: −20 to −17 ms), with a mean bias of −6 ms (range: −7 to −5 ms) (Table 4 and Supplementary Materials Figure S1).

For PDFF, the intra-class correlation for individual technologists on different days was 0.99 (95% CI: 0.98 to 1.00), with a mean difference between the upper and lower limits of agreement of 0% (range: −0.1 to 0%) and −0.8% (range −1 to −0.7%), respectively. The mean upper and lower limits of agreement were 0.8% (range: 0.7 to 0.8%) and −1.1% (range −1.3 to −1.1%), with a mean of −0.2% (range: −0.3 to −0.1%) (Table 4 and Supplementary Materials Figure S1).

## 4. Discussion

Noninvasive tests are growing in popularity to support the management of patients; however, only 3% of FDA-approved AI imaging solutions are implemented in paediatrics [24]. Hence, it is important to investigate and show the utility of NITs in paediatric populations. In this study, we aimed to provide a set of reference values for corrected T1, a standardised multiparametric MRI marker, within a population of nominally healthy participants. Our findings showed that cT1 values of <800 ms and PDFF values of <5% are characteristic of a nominally healthy paediatric population made up of children and young adults. Furthermore, we found that cT1 and PDFF values are comparable in healthy children, young adults and adults without any known chronic liver disease.

Liver disease is a growing clinical problem fuelled, in part, by the rapidly increasing prevalence of obesity and steatotic liver disease (SLD). Unlike neurodegenerative, cardiac or chronic pulmonary diseases, liver disease is particularly challenging to diagnose as it is usually asymptomatic. Although biochemical serum markers are used to support patient management, these markers are not specific and can be normal in the presence of disease [25]. If left unmanaged, the continued progression of liver diseases typically results in the development of advanced fibrosis leading to cirrhosis, the development of primary liver cancers [8], cardiovascular disease [2], liver transplantation [26] and a higher cumulative incidence of overall mortality [2]. Therefore, the early detection of disease is an essential component of preventing adverse clinical outcomes and cannot be based on clinical history and examination alone.

To support early detection, defining normal ranges for new biomarkers is essential if they are to be used to assess the presence, absence or change in disease over time. This is especially so as serological tests in children show low sensitivity and specificity and do not seem to be applicable for screening patients with the risk of progression of liver disease [25]. There is an additional need for objective biomarkers to determine the efficacy of potential treatments, efficacy of planned treatments [27] and prediction of adverse clinical outcomes [23]. Currently, the use of NITs to support adult patient management is an area of active research, with societies such as EASL recently publishing detailed clinical guidance on the utility of NITs for the evaluation of liver disease severity and prognosis [28]. Moreover, the utility of NITs (either individually, in sequence or in combination) for differing contexts of use is being heavily investigated by various consortia such as Liver Investigation: Testing Marker Utility in Steatohepatitis (LITMUS), Non-Invasive Biomarkers of Metabolic Liver Disease (NIMBLE) and NASH Consortium for the Assessment of Non-Invasive Testing in Monitoring Interventions and Treatment Response and Major Liver Related Outcomes (NAIL-NIT). However, the current guideline updates acknowledge that there is a paucity of data showing the validated reference ranges of these tests in paediatric (children and young adult) populations [4].

In this study, we validated the cT1 and PDFF thresholds in paediatric patients and compared these to an adult population. A previous study evaluated the reference ranges for a healthy low-risk adult population and showed that the cT1 values had a median < 800 ms [19]. Age group comparisons showed statistically significant differences between the paediatric and the adult population for cT1; however, these were clinically negligible and fell within the repeatability coefficient [20]. Similarly, statistically significant but clinically negligible differences in PDFF between age groups were observed. There were no statistically significant sex differences in both cT1 and PDFF between young males and females. This suggests that unlike markers like FIB-4, MELD/PELD [29], a correction of cT1 and PDFF for age and sex may not be necessary in paediatric populations in practice.

Multiparametric MRI has been noted as being one of the most promising tools to provide more clinically practical, affordable and accurate non-invasive patient monitoring for children and adolescents with chronic liver disease [30]. Multiple studies have shown both diagnostic and prognostic utility of cT1 in the management of a wide range of paediatric chronic liver diseases. Due to its ability to give a panoramic view of the liver [31], good correlation with histology [13,14], standardisation across different scanners and field strengths, and low inter-observer variability [20], cT1 has been described as a ‘virtual biopsy’ with the potential to inform risk stratification of patients and assist in the decision to withdraw treatment, both of which are pivotal steps in patient management [31]. Furthermore, in addition to supporting clinical decision-making [32] and its proposed use as a substitute for biopsy in patient follow-up [33], by providing additional metrics that can be used to assess disease heterogeneity [34], cT1 can support the long-term monitoring of disease by acting as an independent predictor of fibrosis [13] and disease relapse [35].

Although some studies have reported on healthy populations, there has been no analysis across sex, age and ethnicity validating the cT1 and PDFF healthy reference ranges in paediatric populations. The validation of such biomarkers is important in the paediatric management of chronic liver disease, where liver biopsies are still used serially, despite their drawbacks and associated high rates of complication. This is also important in supporting the assessment of treatment efficacy in pharmacotherapy clinical trials as the Paediatric Research Equity Act (PREA), enforced by regulators like the FDA, states that unless granted a waiver, deferred or deemed inapplicable, pharmacotherapy developers are required to show the safety and effectiveness of their product for the claimed indication(s) in paediatric patients [36].

Although non-standardised tools have shown utility in patient management [37], the use of tests that are standardised across devices allows for the objective evaluation of patients in a device-agnostic manner. Thus, in addition to being standardised across MRI scanners (Siemens, GE and Philips) and field strengths (1.5T and 3T), in terms of technical performance, inter- and intra-rater reproducibility has been reported for an adult population but not for a paediatric population [20]. The findings from this study showed good performance, with tight confidence intervals across individual technologists [20]. This is particularly important when assessing clinical benefit, safety and toxicity during the development of pharmacotherapies, as well as in the general management of patients.

Amongst the strengths of this study, there are some limitations to consider. In all the cohorts pooled together to make up the paediatric sub-group, no Tanner stage information was available except for the METCOG cohort and, thus, we could not ascertain the actual age of puberty onset. Therefore, the onset of puberty was assumed to be at age 13 years for both males and females. We accept the limitations this may have placed on the interpretation of the data; nevertheless, this allowed us to explore any potential changes in cT1 and PDFF that could be associated with puberty. There was a bias in participant sex as the METCOG study was wholly compromised of Hispanic male volunteers. Future global pooled studies of this nature should include more ethnicities alongside bigger participant cohorts (which are adequately powered to assess differences in a wider range of ethnicities and balanced sex groups), the collection of Tanner information and balanced sex groups. Although we endeavoured to include asymptomatic children with no known liver disease in this study, it is possible that some participants had an underlying, undiagnosed, asymptomatic condition. This is particularly relevant as a National Health and Examination Survey carried out between 2005 and 2014 among children with a BMI of less than the 85th percentile found an 8% weighted prevalence of lean MASLD. Therefore, we endeavoured to align with current screening guidelines for MASLD in children by including the assessment of liver function tests alongside BMI [38]. Future studies looking at reference ranges in healthy populations should consider including more comprehensive participant assessments and definitions of ‘healthy’. Regarding technical improvements, unlike in previous studies, where region of interest (ROI)-based analyses from a single slice were analysed to produce cT1 and PDFF maps [19], mpMR methods have been improved over the years and now use automatic segmentation of both maps. Furthermore, unlike in the UKBB, where many imaging techniques had to be implemented in a very short timeframe, resulting in the acquisition of a single slice, the mpMRI protocol now assesses four slices. By embedding redundancy into the data acquisition, not only are sampling errors reduced but the new acquisition ensures that previously highlighted limitations in very heterogeneous disease distributions are addressed [19].

In summary, this study described the reference ranges of cT1 and PDFF values from an individual multi-centre pooled population of nominally healthy children and young adults. The ranges presented here have the potential to serve as a benchmark of normality when assessing various chronic liver diseases with cT1 and PDFF.

## Figures and Tables

**Table 1:** Demographics table of the cohorts included in the pooled participant analysis of healthy individuals. Paediatric participants were aged 6–18 years, whilst adult participants were aged 21–81 years.

Group	Study	Study Identifier	Location	Country	N	Median Age (Years)	Sex (Male)	Ethnicity	BMI (kg/m^2^)
Paediatric	Kids4Life	NCT03198104	Children’s Memorial Health Institute (IPCZD)	Poland	21	15 (4)	38%	Caucasian	20.7 (3.8)
			Southampton Hospital	United Kingdom	27	11 (7)	44%	Caucasian	17.7 (3.1)
	METCOG	Medical Research Council (MR/N029194/1) and CONACyT México (FONCICIT/37/2016)	Hospital Infantil de México Federico Gómez	Mexico	53	8 (2)	100%	Hispanic	15.4 (2.1)
Adult	UKBB	Access application 9914	Multisite UK study	United Kingdom	500	65 (14)	44%	Mixed	23.2 (2.3)
	CoverScan	NCT04369807	Multisite UK study	United Kingdom	71	44 (20)	31%	Mixed	22.4 (3.8)

**Table 2:** Distribution of cT1 and PDFF values for healthy adults and paediatric sub-groups. Paediatric participants were aged 6–18 years, whilst adult participants were aged 21–81 years.

Sub-Group	Percentiles				
	2.5th	25th	Median	75th	97.5th
cT1 (ms)					
Paediatrics	683	725	748	768	820
Adults	654	714	738	754	791
PDFF (%)					
Paediatrics	1	1.3	1.7	2.1	4.4
Adults	0.9	1.8	2.3	3	4.6

**Table 3:** Groupwise distribution of cT1 and PDFF between healthy paediatric and adult sub-groups. Values are reported as median (interquartile range). Paediatric participants were aged 6–18 years, whilst adult participants were aged 21–81 years.

	cT1 (ms)	*p*-Value	PDFF (%)	*p*-Value
Age groupwise comparison				
Paediatric	748 (34)	<0.001	1.9 (0.9)	<0.001
Adult	732 (35)		2.4 (0.9)	
Sex groupwise comparison				
Paediatric				
Female	755 (37)	0.268	2.2 (1.3)	0.452
Male	746 (32)		1.7 (0.6)	
Adult				
Female	735 (35)	0.054	2.3 (0.9)	<0.001
Male	729 (34)		2.6 (0.9)	

**Table 4:** Reproducibility of cT1 and PDFF observations. For inter-class correlations (ICCs), findings are reported as the ICC (95% CI); all other metrics are reported as the mean (range).

Metric	Intra-Class Correlation	Intra-Rater Lower Limit of Agreement	Intra-Rater Bias	Intra-Rater Upper Limit of Agreement	Inter-Rater Lower Limit of Agreement	Inter-Rater Bias	Inter-Rater Upper Limit of Agreement
**cT1**	0.99 (0.99 to 1.00)	−8 ms (−8 to −7 ms)	−1 ms (−2 to 0 ms)	6 ms (5 to 7 ms)	−18 ms (−20 to −17 ms)	−6 ms (−7 to −5 ms)	8 ms (4 to 11 ms)
**PDFF**	0.99 (0.98 to 1.00)	−0.8% (−1 to −0.7%)	0% (−0.1 to 0%)	0.7% (0.7 to 0.7%)	−1.1% (−1.3 to −1%)	−0.2% (−0.3 to −0.1%)	0.8% (0.7 to 0.8%)

## Data Availability

The data and analytic methods used in this study remain the property of the individual study sponsors. All deidentified participant data may be made available to other researchers upon request following permission granting, investigator support and the signing of a data access agreement.

## Supplementary Materials

The following supporting information can be downloaded at https://www.mdpi.com/article/10.3390/children11101230/s1: Figure S1: Bland–Altman plots showing the intra- and inter-rater agreement between operators (technologists) for read 1 (same day) and read 2 (after 30 days) for the assessment of (A) corrected T1 (cT1) and (B) proton density fat fraction (PDFF); Figure S2: Box plots illustrating groupwise distribution of cT1 and PDFF between all investigated age sub-groups. Age groups were defined as follows: Child aged < 13 years, Young Adult aged ≥ 13 years, Adult aged 19–60 years and Older Adult aged > 60 years; Table S1: Groupwise distribution of cT1 and PDFF between age sub-groups. Age groups were defined as follows: Child aged < 13 years, Young Adult aged ≥ 13 years, Adult aged 19–60 years and Older Adult aged > 60 years.
