# Supplementary material for: Reference Range of Quantitative MRI Metrics Corrected T1 and Liver Fat Content in Children and Young Adults: Pooled Participant Analysis
Source: Children (Basel). 2024 Oct 12;11(10):1230. doi: 10.3390/children11101230 (PMC11506660; doi:10.3390/children11101230)
Supplement: Supplementary file 1 [file children-11-01230-s001.zip › Supplementary Figure S1.pdf]

Supplementary Figure S1: Bland-Altman plots showing the intra- and inter-rater agreement between operators (technologists) for read 1 (same day) and read 2 (after 30 days) for the assessment of (A) corrected T1 (cT1) and (B) proton density fat fraction (PDFF).

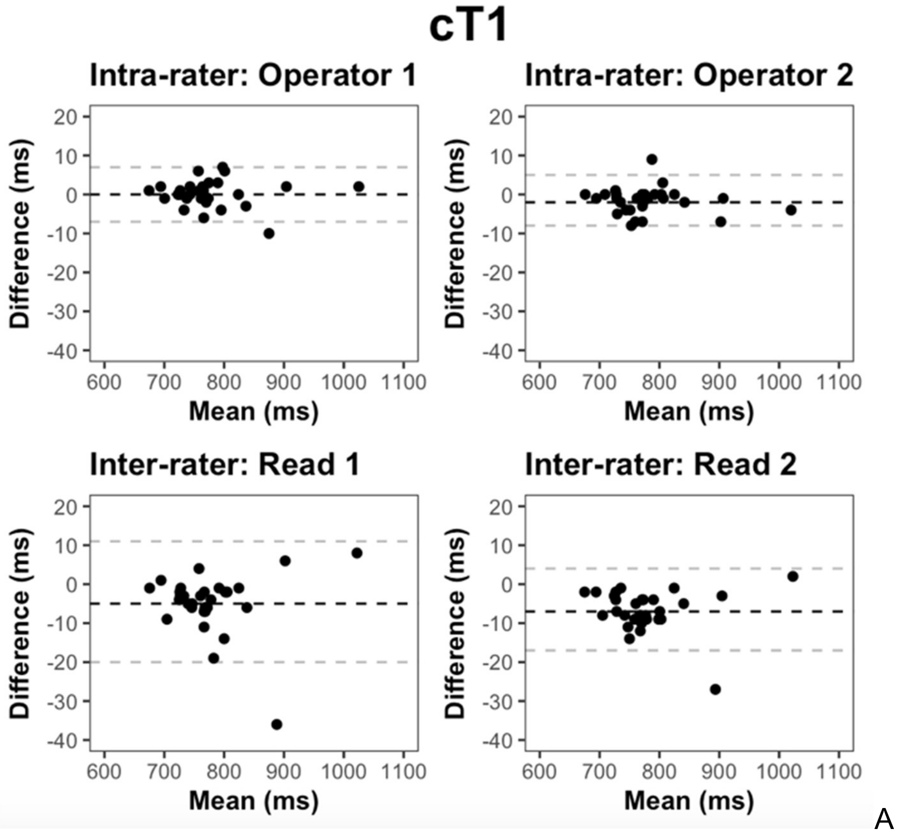

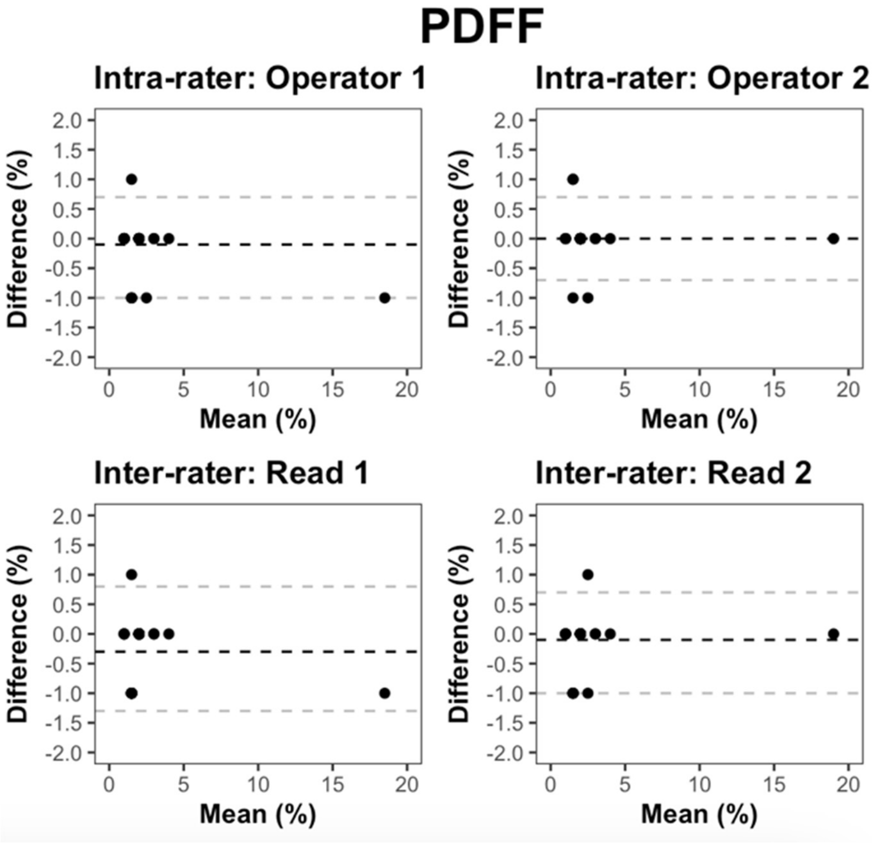

PDFF
Intra-rater: Operator 1
Intra-rater: Operator 2
Inter-rater: Read 1
Inter-rater: Read 2
Difference (%)
Mean (%)

B
