# Supplementary material for: Reference Range of Quantitative MRI Metrics Corrected T1 and Liver Fat Content in Children and Young Adults: Pooled Participant Analysis
Source: Children (Basel). 2024 Oct 12;11(10):1230. doi: 10.3390/children11101230 (PMC11506660; doi:10.3390/children11101230)

Supplementary Figure S2: Box plots illustrating groupwise distribution of cT1 and PDFF between all investigated age sub-groups. Age groups were defined as: Child aged < 13 years, Young Adult aged ≥ 13 years, Adult aged 19-60 years and Older Adult aged > 60 years.

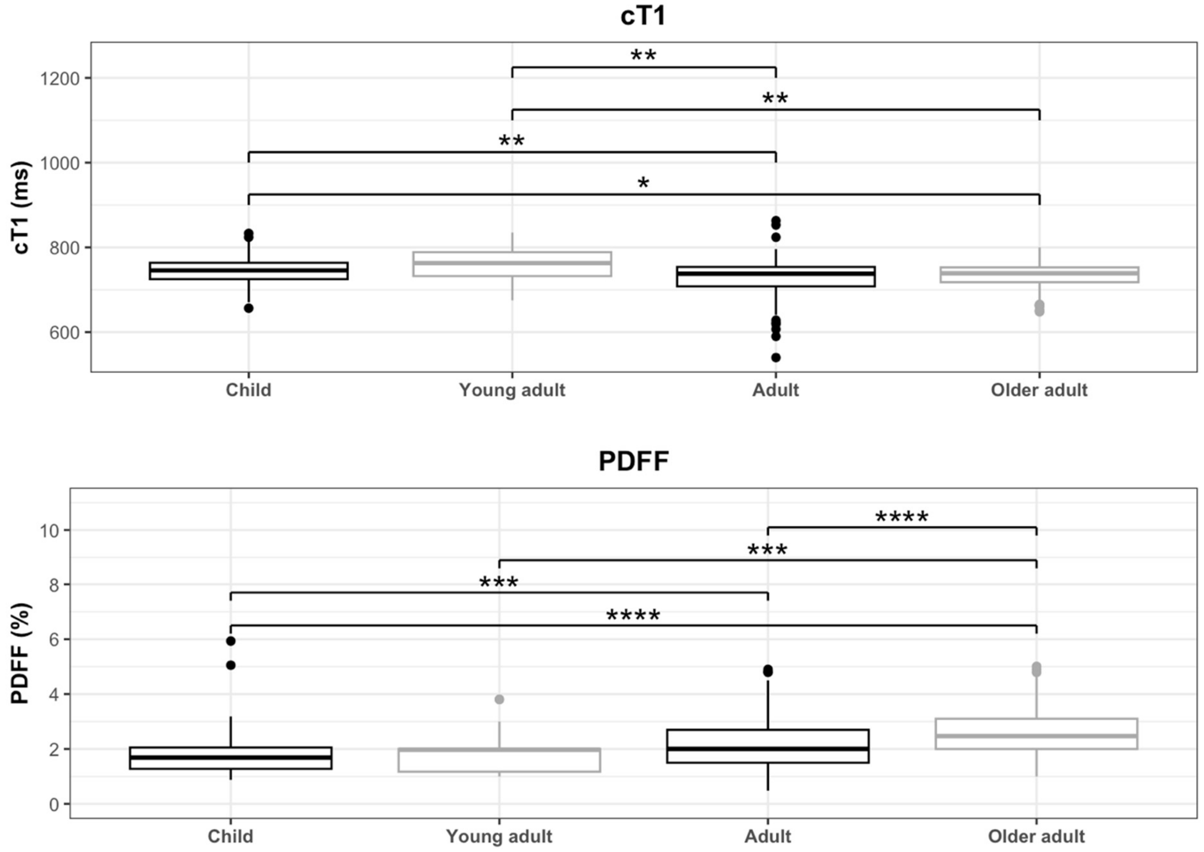

Supplement: Supplementary file 1 [file children-11-01230-s001.zip › Supplementary Figure S2.pdf]
