# Supplementary material for: Reference Range of Quantitative MRI Metrics Corrected T1 and Liver Fat Content in Children and Young Adults: Pooled Participant Analysis
Source: Children (Basel). 2024 Oct 12;11(10):1230. doi: 10.3390/children11101230 (PMC11506660; doi:10.3390/children11101230)
Supplement: Supplementary file 1 [file children-11-01230-s001.zip › Supplementary Table S1.pdf]

Supplementary Table S1: Groupwise distribution of cT1 and PDFF between age sub-groups. Age groups were defined as: Child aged < 13 years, Young Adult aged ≥ 13 years, Adult aged 19-60 years and Older Adult aged > 60 years.

	cT1 (ms)	p-value	PDFF (%)	p-value
**Age group-wise comparison**				
**Child**	746 (39)	0.062	1.7 (0.8)	0.60
**Young adult**	763 (56)		2.0 (0.8)	
**Adult**	738 (46)	0.17	2.0 (1.2)	< 0.001
**Older Adult**	739 (35)		2.5 (1.1)	
**Sex group-wise comparison**				
**Child**				
**Female**	753 (25)	0.35	2.0 (1.1)	0.71
**Male**	744 (39)		1.6 (0.7)	
**Young adult**				
**Female**	758 (68)	0.59	1.9 (0.8)	0.87
**Male**	766 (15)		2.0 (0.8)	
**Adult**				
**Female**	739 (44)	0.019	1.8 (1.0)	< 0.001
**Male**	723 (54)		2.3 (1.2)	
**Older Adult**				
**Female**	742 (32)	0.15	2.3 (1.1)	0.013
**Male**	736 (36)		2.6 (1.2)	
**Ethnicity group-wise comparison**				
**Hispanic**	741 (36)	0.19	1.6 (0.7)	< 0.001
**Caucasian**	739 (41)		2.2 (1.1)	
**Other**	746 (26)		1.8 (1.9)	
